# Perioperative Transthoracic Lung Ultrasound for Assessment of Pulmonary Outcome in Adolescent Idiopathic Scoliosis Patients: Prospective, Observational Pilot Study

**DOI:** 10.1038/s41598-019-54437-y

**Published:** 2019-11-28

**Authors:** Hae Wone Chang, Young Ju Won, Byung Gun Lim, Seung Woo Suh, Dong Kyu Lee, Il Ok Lee, Sul Gi Ji, HeeZoo Kim

**Affiliations:** 1 Department of Anesthesiology and Pain Medicine, Eulji University Hospital, Seoul, Republic of Korea; 2 Department of Anesthesiology and Pain Medicine, Korea University Guro Hospital, Korea University College of Medicine, Gurodong-ro 148, Guro-gu, Seoul, 08308 Republic of Korea; 3 Scoliosis Research Institute, Department of Orthopedics, Korea University Guro Hospital, Korea University College of Medicine, 148 Gurodong-ro, Guro-gu, Seoul, 08308 Republic of Korea

**Keywords:** Adaptive clinical trial, Outcomes research

## Abstract

The purpose of study was to evaluate the perioperative lung ultrasound findings of patients undergoing scoliosis correction. LUS examination was performed examined three 3 times for each patient: 20 min after starting mechanical ventilation of the lungs(preoperative), after surgery when the patient was placed in the supine position(postoperative), and 20 min after arrival in the post-anaesthesia care unit. Arterial blood gas analyses, mechanical ventilation parameters, peripheral oxygen saturation(SpO2) were also checked. Twenty-six patients completed the study. The changes of LUS score(20 min) was significantly negatively correlated with the partial pressure of arterial oxygen(PaO2)/fraction of inspired oxygen(FiO2) ratio change(P = 0.039, r = −0.40). The change in mean convex side LUS score was significantly greater than that of the concave side as determined by two-factor repeated measures analysis of variance(p = 0.001). Multiple regression analysis revealed perioperative LUS change was the significant factor related to the oxygen index change (p = 0.042). One case of pneumothorax was diagnosed and pleural thickening more than 5 mm was detected in 8 patients and five patients of those were diagnosed pleural effusion and performed thoracentesis after surgery. Postoperative increase of LUS score was related with deteriorating of oxygenation at one day after surgery, and it suggests that lung ultrasound allows prediction of postoperative hypoxia and facilitates the diagnosis of pulmonary complications at operation room in AIS patients.

## Introduction

Scoliosis correction surgery for adolescent idiopathic scoliosis (AIS) is effective in preventing deterioration of lung function caused by disease progression and improving lung volume over the long term^1–3^. However, complications of the respiratory system and pulmonary dysfunction may occur in the immediate postoperative period^4,5^. General anaesthesia (GA) and positive pressure ventilation can cause decreased lung volumes, expiratory flow rates, and oxygenation after surgery as a result of positive pressure ventilation, partial recovery of respiratory muscle, pain, and immobilization^6,7^.

Respiratory complications in the perioperative period constitute a major cause of postoperative morbidity after scoliosis surgery. Acute respiratory failure is reported in 3.47% of patients undergoing corrective surgery for scoliosis, and reintubation is reported in 1.27% of cases^8^. Moreover, pulmonary function test (PFT) values decrease by 60% during the immediate postoperative period^4^. PFT values remain significantly depressed for 1 week after surgery, at half the preoperative baseline values^4^.

It has been reported that lung ultrasound (LUS) at a patient’s bedside immediately following surgery can be useful for diagnosing respiratory complications^9,10^. LUS has proven to be a valuable bedside diagnostic tool for pneumothorax, with high sensitivity and specificity (78.6% and 98.4%, respectively), and a higher rate of detecting abnormalities such as lung alveolar consolidation and pleural effusion than bedside chest X-ray or physical examination^11–13^. LUS has also been used to diagnose anaesthesia-induced atelectasis in paediatric patients^14,15^. Audrey and colleagues suggested use of a modified LUS score in adult patients and showed that the LUS score correlates with changes in oxygenation during the perioperative period^9^.

Previous studies on perioperative respiratory monitoring of AIS patients have focused on evaluating the prevalence of postoperative pulmonary complications such as reintubation or postoperative pleural effusion. There is no study that uses LUS to evaluate aeration loss caused by lung deterioration during the perioperative period.

This study aimed to use transthoracic lung ultrasound (LUS) to correlate the differences in preoperative LUS versus postoperative LUS with changes in the oxygenation status (PaO_2_/FiO_2_ ratios) during perioperative period.

## Materials and Methods

### Patients and study protocol

This was an investigator-initiated, prospective, observational study. The use of transthoracic lung ultrasound during general anesthesia and postoperative period was approved and followed as part of clinical management accordance with regulation and guideline of Institutional Review Board protocol (Korea University IRB No. 2017GR0131). The trial was registered in the UMIN clinical trials registry (unique trial number: UMIN000028449; registration number: R000032565 Principal investigator: Young Ju Won, Date of Registration: 04 August 2017). After tiral registration, all patients were recruited from the Department of Orthopaedic Surgery, Korea University Guro Hospital. Written informed consent for the use of image of LUS and perioperative data was obtained from all parents of the patient under age 19 and /or patients in case of patient age over 19. Patients scheduled to undergo elective scoliosis correction surgery, aged 13 to 22 years, were enrolled in the study at admission to the hospital the day before surgery. Patients who refused to undergo pulmonary ultrasound, refused to participate in the study, were hemodynamically unstable before surgery, had body mass index >30 kg m^−2^, had history of intrathoracic procedures, had severe pulmonary disease (forced expiratory volume in 1 s less than 30% of predicted value), or for whom artery cannulation could not be performed were excluded. After collected of data, we registered in UMIN- IDCR(individual case data repository, https://upload.umin.ac.jp/cgi-bin/icdr_e/ctr_view.cgi?recptno = R000032565).

### Anaesthetic protocol

All patients underwent GA using total intravenous anaesthesia (TIVA) with continuous infusion of propofol and remifentanil. When induction of general anesthesia was completed, standardized mechanical ventilation was maintained (GE Datex-Ohmeda Aestiva 3000; GE Healthcare, Wauwatosa, WI, USA) for all patients. Volume-controlled ventilation with tidal volume of 8 mL kg^−1^, inspiratory to expiratory ratio of 1:2, and positive end expiratory pressure (PEEP) of 5 cm H_2_O was implemented. Ventilation frequency was adjusted to maintain end-tidal CO_2_ partial pressure of 30–35 mm Hg. Anaesthesia was maintained and continuously adjusted with an oxygen-air mixture of 0.5 inspired oxygen fraction.General anaesthesia was maintained until the postoperative LUS exam. After the patient recovered spontaneous breathing and consciousness, extubation was performed and the patient was transferred to the post-anaesthetic care unit (PACU). After discharge from the PACU, patients were monitored in the high-care unit postoperatively for 24 h.

### Surgical technique

All patients were operated in prone position with posterior- only approach. During Minimal invasive surgery, all vertebrae were instrumented to form an all-pedicle-screw construct. All the appropriately sized screws are put through minimal incisions by mobilizing skin and soft-tissue flaps in the same manner. On the other hands, in process of open surgery, the surgeon dissected spine subperiosteally up to the tips of transverse processes of vertebrae at all levels. After all the pedicle screws have been introduced, two rods are inserted to correct most of the deformity. Short apical rib resection thoracoplasty was performed both MIS and open surgery. It was done using 5 mm burr for kyphoscoliosis that affects humped ribs on convex side.

### LUS and data collection

**Figure 1:**
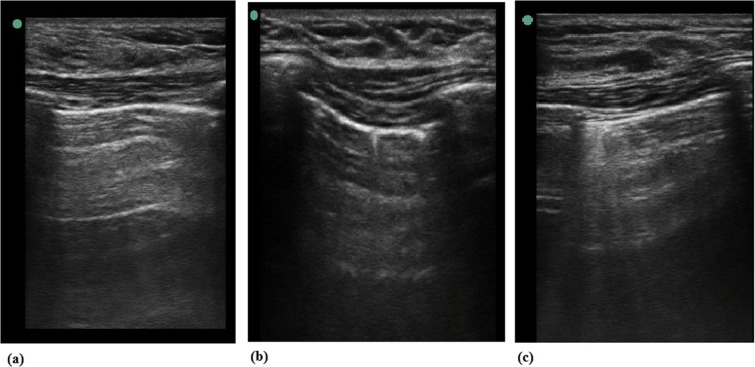
Transthoracic lung ultrasound (LUS) score image (**a**) Modified lung ultrasound scores 0, (**b**) Modified lung ultrasound scores 1, (**c**) Modified lung ultrasound scores 2.

All ultrasound scans were performed by the same two anaesthetists, who had experience with more than 100 cases in which LUS scans were performed in adults using a Sonosite Edge ultrasound system (Fuji Film, Bothell, WA, USA) and 6–12 MHz linear probe. The default scan depth was set at 4.9 cm and if B-lines was suspected, we increased depth to rule out artifacts including Z-line which is known to fade out at a lesser depth. LUS examination was performed at three time points for each patient: 20 min after starting mechanical ventilation of the lungs before patients were placed in the prone position (preoperative), after surgery at the time the patient was placed in the supine position (postoperative), and at 20 min after arrival to the PACU. After Intubation and extubation, we performed ABGA and Lung ultrasound examination after 20 mins because of patient’s safety during data acquisition, and lung atelectasis change of ventilation change needs 20 min^16^. Patients were scanned in the supine position following the LUS examination method described by Monastesse and colleagues (Fig. 1)^9^. The thorax was divided by the anterior axillary line, the posterior axillary line, and a horizontal line beneath nipple. Twelve intercostal spaces of each area were scanned and analysed. Aeration loss was assessed by calculating the modified LUS score that is calculated mainly using the amount of B-lines (Table 1). Two lung ultrasound examiners scored each area after simultaneously examination of the lung scan respectively and if it was not consistent, re-evaluate and have a consensus. The LUS score of the hemithorax (0–18) was then calculated by adding the six individual quadrant scores of each hemithorax, with higher scores indicating more severe aeration loss. Demographic and surgical data such as age, sex, surgery duration, anaesthesia duration, preoperative serum albumin level, postoperative serum albumin level, intraoperative blood loss, and volume of intraoperative intravenous fluid administrated were collected. Respiratory status data were collected at each study point under GA, including arterial blood gas analysis, mechanical ventilation parameters [tidal volume, respiratory frequency, PEEP, and fraction of inspired oxygen (FiO_2_)], peak inspiratory pressure, end-inspiratory plateau pressure, peripheral oxygen saturation (SpO_2_), partial pressure of arterial oxygen (*P*aO_2_) and estimated delivered FiO_2_. The presence and severity of pain were assessed by a clinician at the recovery room, by using an 11-point (0–10) numeric rating scale. Arterial blood gas analysis was performed pre-incision after general anaesthesia, between skin suture and extubation, and at postoperative day 1. Pleural effusion was diagnosed when hypoechoic interpleural fluid was greater than 10 mm in each lung sonography lesion, bounded at the surface by a straight line in the parietal layer^17,18^. Identification of the lung behind the pleural effusion was necessary.

**Table 1:** Modified lung ultrasound scores.

LUS score of 12 individual pulmonary quadrant			
0	1	2	3
0–2 B-lines	≧ B-lines OR one or multiple small subpleural consolidations separated by a normal pleural line	Multiple coalescent B-lines OR multiple small sub- pleural consolidations separated by a thickened or irregular pleural line	Consolidation OR Small subpleural consolidation of >1 × 2 cm in diameter

### Statistical analysis

The sample size calculation was based on the previous study^9^, the association between differences in LUS scores (preoperative versus postoperative) and change in partial pressure of arterial oxygen (PaO_2_)/FiO_2_ ratio. Assuming that the correlation between the two factors was 0.45, a sample size for the group was determined to be 29, as calculated by correlation: bivariate normal model with exact distribution, a level of significance of 0.05, and a power of 0.8. The sample size for enrolment was 29.

Statistical analysis was performed with GraphPad Prism version 7.03 for Window (GraphPad Inc., La Jolla, CA, USA). Results are expressed as mean (standard deviation) and median (range) or number of patients (%), as appropriate. Pearson’s correlation was used to assess the relationship between differences in LUS scores (preoperative versus postoperative, preoperative versus PACU) and differences in pre- and postoperative variables, including change in partial pressure of arterial oxygen (*P*aO_2_)/FiO_2_ ratio. Spearman’s correlation coefficient was used to determine whether there were significant relationships between *P*aO_2_/FiO_2_ ratios or LUS scores and surgical or patient variables. Multiple regression analysis was assessed to predict the degree of changes in the O2 index using independent variables including age, gender, Cobb’s angle, body mass index and LUS changes from preoperative to postoperative period. Comparisons between convex side LUS and concave side LUS score changes were analysed using a two-factor repeated measured analysis of variance (ANOVA) with a Bonferroni correction after D’Agostino-Pearson normality test. Spherocity was not assumed, and the Geisser-Greenhouse correction was applied. The overall alpha level was set at 0.05 with a Bonferroni adjustment to control for multiple significant tests; thus, alpha was set at 0.017 to determine significance of individual tests.

## Results

Between August 2017 and March 2018, 29 AIS patients who underwent scoliosis correction surgery were examined using LUS. All scoliotic curves were convex to the right. Patients’ baseline characteristics and surgery parameters are summarized in Table 2. Twenty-two patients underwent minimally invasive spine surgery (MISS), five patient underwent MISS only, one patient underwent open correction, and one patient underwent open correction with right thoracoplasty. During the study, one patient developed a right-side pneumothorax after surgery, so it was impossible to evaluate the LUS score after surgery (postoperative and PACU time points). Two patients presented unstable blood pressures and transferred to the intensive care unit. Therefore, a total of three patients were dropped from the study. Twenty-six patients completed the study.

**Table 2:** Patient characteristics and surgical factors.

Variables (N = 29)	Mean (SD) or number (%)
Age	17.7 ± 4.1
Sex (Male/Female)	2(6.9%)/27(93.1%)
Height	159.6 ± 8.6
Weight	50.4 ± 8.8
Preoperative vital capacity (%)	74.0 ± 16.3
BMI	
Preoperative serum albumin (g/dL)	4.4 ± 0.3
MISS right thoracoplasty	22
Open thoracoplasty	1
MISS	5
Open correction	1
Postoperative serum albumin (g/dL)	3.1 ± 0.3
Anaesthesia duration (min)	440.6 ± 71.5
Surgery duration (min)	326.9 ± 69.3
Cobb’s angle (°)	57.2 ± 12.4
Total fluid intake (ml)	2360.3 ± 734.4
Total urine output (ml)	844.0 ± 369.9
Total.blood.loss (ml)	731.0 ± 514.2
Preoperative P_peak_ (cmH_2_O)	18.9 ± 2.0
Preoperative P_plateau_ (cmH_2_O)	17.6 ± 2.6
Preoperative TV (ml)	412.1 ± 70.5
Postoperative P_peak_ (cmH_2_O)	21.4 ± 1.8
Postoperative P_plateau_ (cmH_2_O)	19.8 ± 2.6
Postoperative OP TV (ml)	406.6 ± 68.0
Preoperative PCO_2_(mmHg)	33.5 ± 2.7
Postoperative PCO_2_ PACU (mmHg)	34.4 ± 3.3
Postoperative PCO_2_ (24 h) (mmHg)	40.3 ± 5.1

BMI, body mass index; MISS, minimally invasive spinal surgery; PCO_2,_ partial pressure of CO_2_; SD, standard deviation; Ppeak, peak airway pressure Pplateau plateau pressure TV, tidal volume.

**Figure 2:**
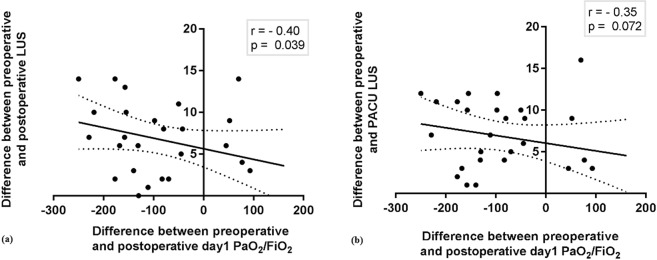
Relationship between changes in the modified LUS scores and changes in the O_2_ index using Pearson correlation coefficients: (**a**) O_2_ index, PaO2/FiO2 ratio, difference between preoperative and postoperative LUS, (**b**) difference between preoperative and PACU LUS. FiO2: fraction of inspired oxygen; LUS: lung ultrasound; PACU: post-anaesthesia care unit; PaO_2_: partial pressure of arterial oxygen.

As shown in Table 3 and Fig. 2, differences in preoperative LUS versus postoperative LUS were significantly negatively correlated with changes in the *P*aO_2_/FiO_2_ ratios from preoperative versus postoperative day 1 (P = 0.039, r = −0.40) and were moderately negatively correlated with the *P*aO_2_/FiO_2_ ratios at postoperative day 1 (*P* = 0.0726, r = −0.35). Cobb’s angle was significantly correlated with total preoperative, postoperative, and PACU LUS scores (*P* = 0.0187, 0.0481, and 0.0248, respectively), but was not correlated with postoperative *P*aO_2_/FiO_2_ ratios or with *P*aO_2_/FiO_2_ ratios at postoperative day 1 (*P* = 0.8321 and *P* = 0.5364, respectively). None of the other factors such as BMI, Cobb’s angle, peri-operative serum albumin, surgery time, anaesthesia time, total fluid intake, operation spine level, thoracoplasty level, preoperative pulmonary vital capacity, total blood loss, time to hospital discharge day, that were thought to be associated with changes in *P*aO_2_/FiO_2_ was significantly correlated. In the multiple regression analysis, LUS changes from preoperative to postoperative period was the significant predictor related to the changes of O2 index (Table 4).

**Table 3:** The correlation between Change of PaO_2_/FiO_2_ and various factors by using Pearson’s correlation coefficient (N = 26).

	Change of PaO_2_/FiO_2_ pre op- post op 24 hr	
	Pearson r	P value
BMI	0.06	0.772
Cobb’s angle	−0.10	0.619
Preop serum albumin	−0.14	0.498
postop serum albumin	−0.12	0.565
Operation time	−0.06	0.766
Anaesthesia time	−0.10	0.605
Total fluid intake	−0.20	0.326
Op level value	−0.10	0.628
Thoracic level	−0.04	0.882
Change of LUSprepost	−0.40	0.039
Change of LUSprepacu	−0.35	0.072
Preop vital capacity (L)	0.02	0.934
Total blood loss (ml)	0.14	0.487
Total urine output	0.07	0.724
P_plateau_	−0.30	0.232
P _peak_	−0.30	0.206
PACU 20RR	0.09	0.663
Post24hPCO_2_	−0.46	0.016
Time to hospital discharge	0.09	0.655

BMI, body mass index; preop,pre-operative; postop, post-operative; Total fluid intake, interaoperative administrated fluid amount; Op level value, the number of corrected spine vertebral level; Thoracic level, the number of rib resection; Change of LUSprepost, difference between preoperative and postoperative LUS; Change of LUSprepacu, difference between preoperative and PACU LUS; Ppeak, peak airway pressure; Pplateau, plateau pressure; Post24hPCO2, partial pressure of CO2 at 24 hours after discharge of PACU;

**Table 4:** Multiple regression analysis of factors related to the changes in the O2 index (difference between the preoperative and postoperative day 1).

Independent variables	B	Beta	t	p	VIF
(Constant)	−179.868		−1.250	0.222	
Body mass index	6.836	0.171	0.910	0.371	1.083
LUS changes (pre- to postoperative)	−0.8.838	−0.403	−2.143 *	0.042	1.083

Adjusted R^2 = 0.088, Durbin-Watson: 1.887. Excluded variables: age, gender, Cobb’s angle.

**Figure 3:**
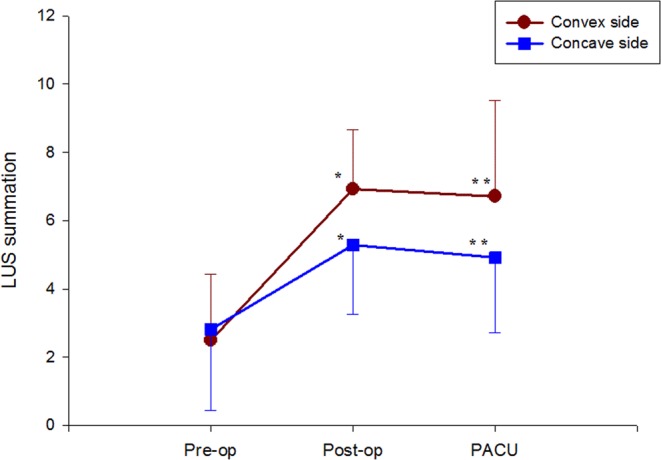
Change in convex side and concave side total modified lung ultrasound scores (LUS) during the perioperative period. Data are presented as mean ± standard deviation. *P < 0.05, **P < 0.01 for total convex side versus concave side LUS at each time point.

The change in mean LUS score on the convex side was significantly greater than that on the concave side as determined by two-factor repeated measured ANOVA (P = 0.001, Fig. 3). Mean LUS scores of the convex side and concave sides at the preoperative measurement were 2.46 ± 0.39 and 2.92 ± 0.48, respectively; there was no difference (n = 26). After surgery, the LUS scores of the convex and concave sides increased significantly at both the postoperative (7.00 ± 1.775 and 5.42 ± 2.04, P < 0.01) and PACU time points (7.23 ± 2.16and 5.31 ± 1.78, P < 0.01). The mean difference between the concave side (right side) and convex side (left side) also increased significantly at the postoperative and PACU time points [means: 1.64 and 1.79; 95% confidence interval (CI): 0.61 to 2.68 and 0.75 to 2.82, respectively].

**Figure 4:**
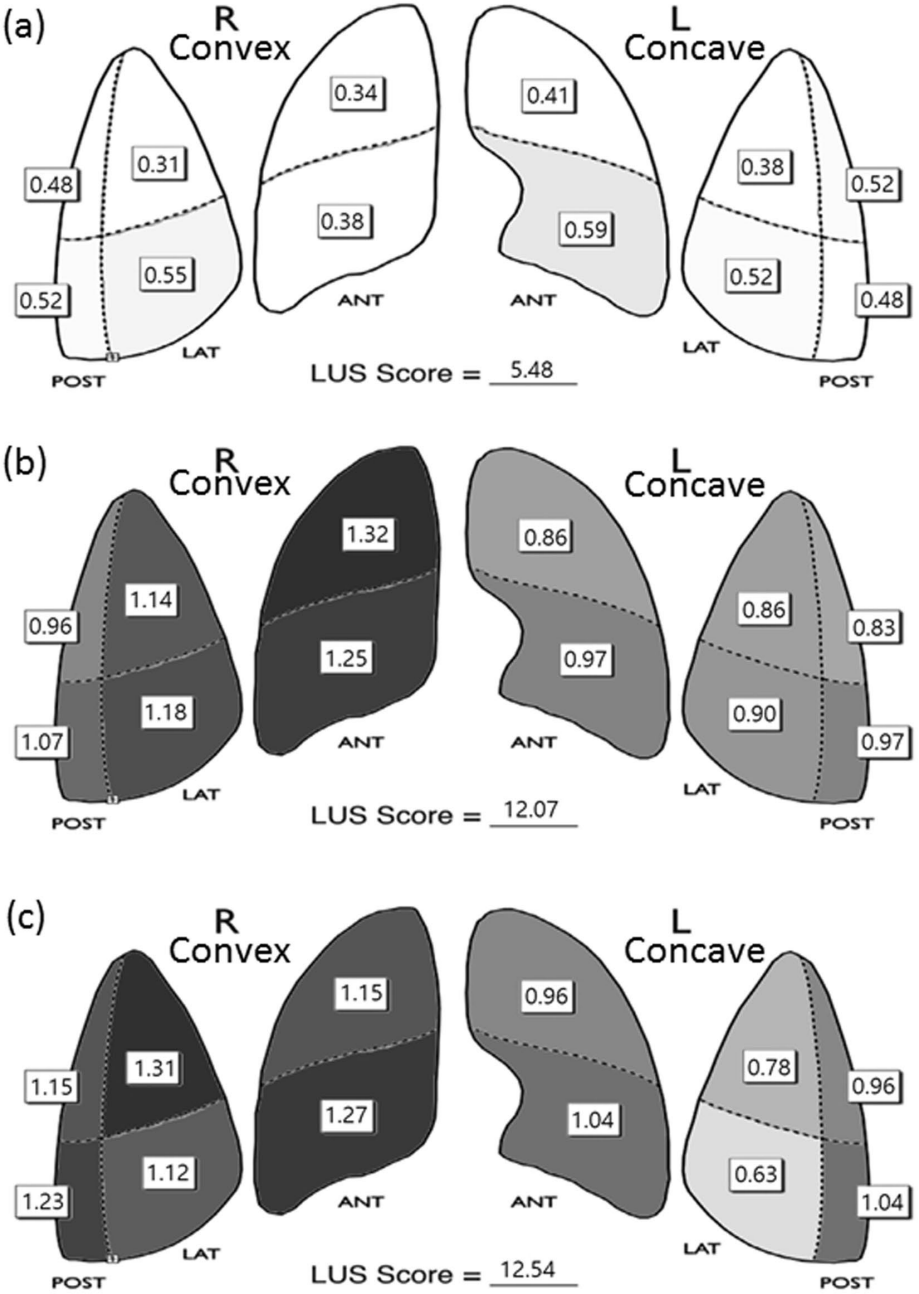
Visual model of LUS results. (**a**) Preoperative LUS, (**b**) postoperative LUS, (**c**) LUS at post anesthetic care unit(PACU); R, Right lung; L Left lung. Note: An LUS value of 0.5 or less was set to white and a value of 1.5 or more was set to black. Between 0.5 and 1.5, when the LUS value was closer to 0.5, the color was brighter, and when it was closer to 1.5, the color was darker. Therefore, a brighter color means a lower LUS, which is preferable. LUS, lung ultrasound score.

Figure 4 shows a visual model of the LUS results. Compared to the concave side of the lung, the convex side of lung showed more dark-colored compartments (the darkness or brightness of the color corresponds to the LUS) in the postoperative and PACU periods (Fig. 4).

There was no instance of desaturation or hyperventilation, deep sedation, agitation, or severe uncontrolled postoperative surgical pain during the PACU stay. Two patients exhibited severe atelectasis on total LUS images at the postoperative time point. After 4 cycles of 5 s 30 cm H_2_O continuous PEEP was administered, we observed improved LUS images, they were extubated and had no adverse event afterall.Oxygen supplied to sixteen patients at postoperative 1 day because of hypoxia or dyspnoea. There were no diagnoses of pleural effusion within the study evaluation period, but pleural thickening more than 5 mm was detected in 8 patients and five patients of those were diagnosed with convex side pleural effusion and performed thoracentesis after surgery.

## Discussion

Our present observational study demonstrated several points. Firstly, postoperative nasal prong oxygen supply was applied to sixteen patients of 26 patients due to hypoxia or symptom of dyspnoea. So we checked the correlation between many factors (Table 3) and PaO_2_/FiO_2_ ratios at postoperative day 1, and LUS score after scoliosis correction surgery was significantly correlated with PaO_2_/FiO_2_ ratios at postoperative day 1. We found that sharp increases in total preoperative and postoperative LUS values resulted in further reduction of *P*aO_2_/FiO_2_ ratios in the postoperative period. Our results suggest that perioperative LUS examination may be used to predict postoperative oxygenation. Therefore, the change in total preoperative and postoperative LUS appears to predict a rapid change postoperative *P*O_2_.

We also found that a change in total preoperative-postoperative LUS score during the perioperative period may be correlated with *P*aO_2_/FiO_2_ on postoperative day 1. Previous articles have focused on the relationship between preoperative PFT and perioperative respiratory deterioration. However, our clinical study showed no correlation between preoperative vital capacity and change in perioperative LUS score or postoperative *P*aO_2_/FiO_2_. This demonstrates that LUS is only useful as a predictor of oxygen saturation during the immediate postoperative period. LUS visualizes the changes in lung atelectasis and lung oedema caused by multiple surgical factors such as surgical level, thoracoplasty level, and intraoperative bleeding, as well as anaesthetic factors such as anaesthesia duration and intake/output fluid balance, thus making it possible to predict the degradation of lung oxygenation ability in the immediate postoperative period^9,19^. Therefore, perioperative LUS is recommended as a useful tool to determine the oxygenation level during the immediate postoperative period.

Secondly, the degree of aeration loss, as evaluated by the transthoracic LUS score, was greater for the convex side lung area than for the concave side lung area during scoliosis correction surgery in AIS patients. Correction of thoracic spine deformity by posterior pedicle instrumentation and rod rotation technique may affect the relative aeration loss in the convex side of the lung in scoliosis. Moreover, 23 patients underwent thoracoplasty of the convex side of the rib hump, which can worsen convex lung aeration loss caused by pain or pleural irritation. Thoracoplasty was thought to be a major factor in postoperative pulmonary complications such as pulmonary function impairment (decreased PFT), requirement for postoperative ventilator support, atelectasis, pneumothorax, pneumonia, and hypoxemia^20,21^.

Chun and others reported that the lung volume of the convex side increases with increasing Cobbs’ angle in AIS patients as compared with the concave side when the preoperative Cobb’s angle is more than 40°^22^. Patients compensated in this manner until corrective operation appeared to have deteriorated lung function and oxygenation disorders due to aeration loss of the convex lung immediately after the surgery. Yuan and colleagues reported these changes, but PFT does not demonstrate a distinction between convex lung and concave lung aeration due to the nature of the test; it shows total lung status and respiratory muscle function status^4^. However, our study was able to demonstrate a specific postoperative lung condition because the six individual quadrant scores of each hemithorax were separately evaluated. Recent studies have shown that the severity of atelectasis of the lung as evaluated by LUS score is highly correlated with the atelectasis volume on computed tomography and changes in oxygenation^9,19^.

Moreover, we observed one case of small pneumothorax in the PACU, which led to early treatment by virtue of the early diagnosis. We observed two cases of severe atelectasis at the postoperative LUS examination, and we performed an alveolar recruitment manoeuvre guided by LUS. The patients were discharged without incident. And although, there were no diagnoses of pleural effusion within the study evaluation period, pleural thickening more than 5 mm was detected in 8 patients and five patients of those were diagnosed with convex side pleural effusion and performed thoracentesis after surgery. These adverse events do not represent statistical significance in our study, where the incidence was low and the sample size was small. However, LUS appears to be useful for quickly detecting and treating these cases.

There are some limitations to this study. Since LUS was performed only during the perioperative period and during the PACU stay, it was not effective in detecting delayed postoperative pleural effusion. Hayashi and colleagues showed that pleural fluid collection induced by pleural irritation was detected at 1 week post-surgery in 71% of scoliosis surgery patents^5^. Moreover, in our centre, thoracoplasty was performed using the short apical rib resections thoracoplasty technique, which has been proven to reduce damage to structures around the rib head^23^. This may, however, lead to delayed postoperative pleural effusion because of reduced prevalence of immediate postoperative pleural effusion induced by vessel injury and bone bleeding.

Another limitation is that the sample size was not large. Further study is recommended to evaluate the prevalence of such adverse events and the sensitivity and specificity of diagnosis by LUS. Since LUS was performed only during the perioperative period and during the PACU stay, further studies are needed to evaluate the benefits of longer perioperative LUS assessment.

In conclusion, a change in aeration loss in the convex side lung area affected the difference in total summed LUS score, which correlated with a change in *P*aO_2_/FiO_2_, suggesting that perioperative LUS examination may be predictive of postoperative oxygenation. Therefore, changes in summed preoperative and postoperative LUS appear to predict rapid changes in postoperative *P*O_2_. Moreover LUS is able to facilitate the diagnosis of pulmonary complications at operation room in AIS patients undergoing corrective surgery. Further study should be assessed for large number of patients, and evaluate postoperative period LUS score versus the correction rate and postoperative complications, especially patients with severe deformity.
